# Gastroduodenal Sarcoidosis With Concomitant Cytomegalovirus Gastritis

**DOI:** 10.14309/crj.0000000000000394

**Published:** 2020-06-11

**Authors:** Kristel Leung, Usman Khan, Iris Teo, Paul James, Jeffrey McCurdy

**Affiliations:** 1 Division of Gastroenterology, Department of Medicine, University of Toronto, Toronto, ON; 2 Division of General Surgery, Department of Surgery, University of Toronto, Toronto, ON; 3 Pathology and Laboratory Medicine, University of Ottawa, Ottawa, ON; 4 Division of Gastroenterology, Department of Medicine, University of Ottawa, Ottawa, ON

## Abstract

Gastrointestinal sarcoidosis in the absence of pulmonary disease is rare. Likewise, cytomegalovirus (CMV) reactivation in the stomach is also rare. We present a 67-year-old woman with symptomatic CMV gastritis and gastroduodenal sarcoidosis who presented with epigastric pain, nausea, and vomiting. Initial gastric biopsies revealed CMV gastritis. Repeat assessment demonstrated worsening disease requiring antiviral treatment. After this, further investigations into ongoing epigastric pain demonstrated noncaseating granulomas on repeat gastrointestinal biopsies. A diagnosis of sarcoidosis was established and treated with prednisone to resolution.

## INTRODUCTION

Sarcoidosis is an uncommon disease of unknown etiology defined by noncaseating granulomas on pathology. Management of sarcoidosis involves assessment for multiorgan disease including pulmonary and cardiac evaluation, with initial treatment involving corticosteroids.^1^ Cytomegalovirus (CMV) reactivation in the gastrointestinal tract is rarely seen outside of the classic immunocompromised population, with diagnosis through CMV inclusion bodies on pathology, CMV antigen positivity with immunohistochemistry, or high serum CMV DNA levels.^2,3^ Treatment options include valganciclovir, ganciclovir, or foscarnet.^4–6^ The low incidence and clinical overlap with multiple diseases are postulated to contribute to delay in diagnosing these patients.^7,8^

## CASE REPORT

A 67-year-old woman of Middle Eastern origin presented with acute on chronic diffuse abdominal pain associated with nausea and vomiting. Her medical history was significant for cirrhosis (Child-Pugh Class A) initially suspected to be secondary to nonalcoholic steatohepatitis in the context of obesity, hyperlipidemia, and liver nodularity on computed tomography (CT), as well as mild pancytopenia, gastroesophageal reflux disease, previous *Helicobacter pylori* infection, remote cholecystectomy, a remote 10 pack-year smoking history, and hyperlipidemia. Her symptoms had not improved with acid suppression or gastric emptying agents. She denied B symptoms, extraintestinal manifestations of inflammatory bowel disease, changes in diet, or recent travel. Medications at the time of presentation were metformin, domperidone, and pantoprazole. There was no contributing family history. Physical examination demonstrated mild epigastric pain without organomegaly or masses, with the remainder of the examination being unremarkable. No rashes or joint abnormalities were observed on examination.

Complete blood count revealed mild pancytopenia: white blood count of 3.2 × 10^9^/L, hemoglobin of 105 g/L, mean cell volume of 80.2 fL, and platelets of 88 × 10^9^/L. Remaining blood work was as follows: aspartate aminotransferase of 45 U/L, alanine aminotransferase of 54 U/L, alkaline phosphatase of 150 U/L, γ-glutamyl transferase of 26 U/L, total bilirubin of 12 μmol/L, albumin of 24 g/L, and international normalized ratio of 1.2. Previous investigations for cirrhosis and autoimmune enteropathies included negative hepatitis C serology, antihepatitis A virus immunoglobulin M (IgM), antinuclear antibodies, antineutrophil cytoplasmic antibodies, antismooth muscle antibody, antienterocyte antibodies, antigoblet cell antibodies, antitransglutaminase antibodies, and antiparietal antibodies. Furthermore, human immunodeficiency virus serologies were negative. Antimitochondrial antibody was intermittently positive with low titers. There was diffuse polyclonal hypergammaglobulinemia on serum protein electrophoresis without abnormal bands. Abdominal ultrasound demonstrated mild ascites and no splanchnic vein thromboses.

**Figure 1.**
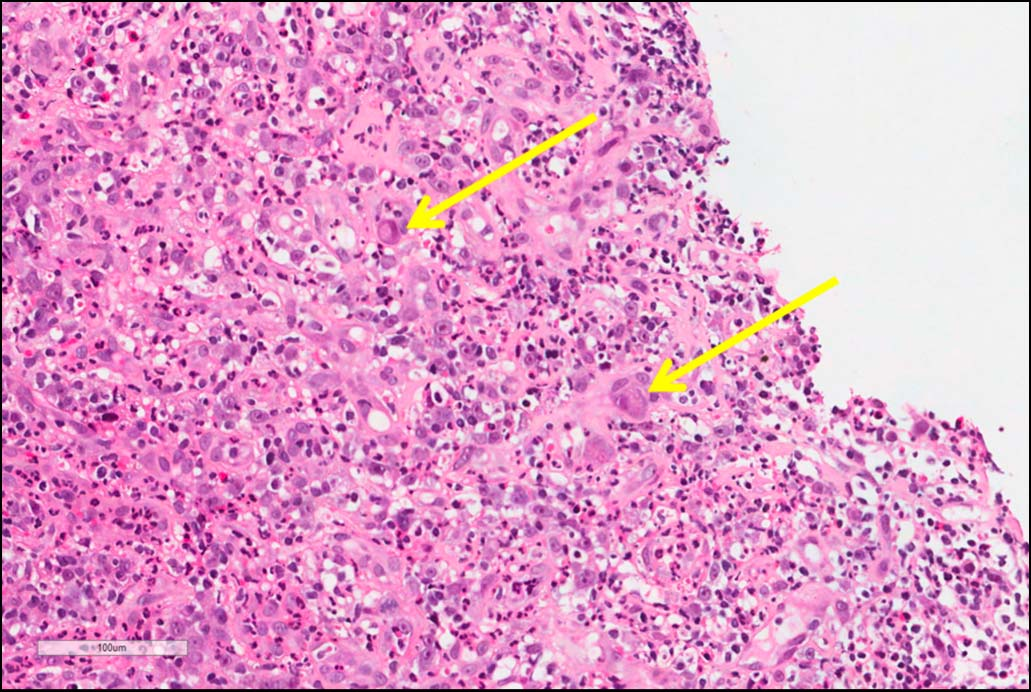
Enlarged endothelial cells with typical cytoplasmic and nuclear inclusions (yellow arrows) in a background of ulcer with extensive neutrophilic inflammation, typical of cytomegalovirus gastritis.

**Figure 2.**
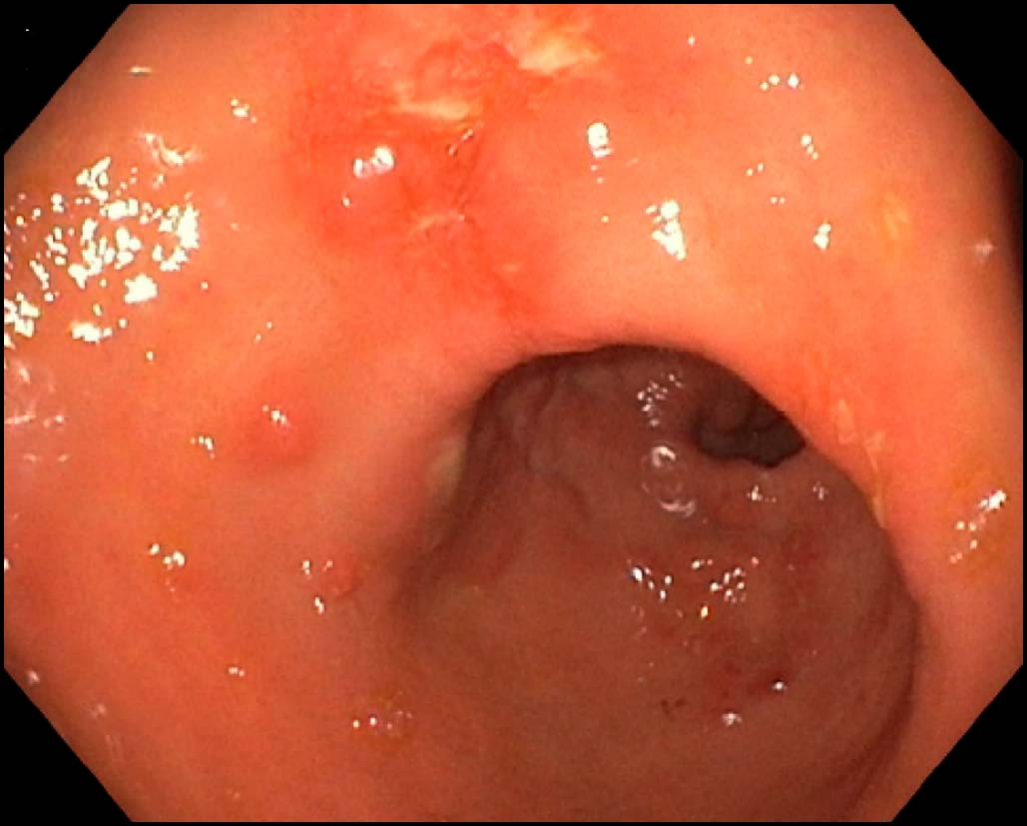
(A) Gastric nodule in the fundus with surrounding inflammation.

Initial esophagogastroduodenoscopy (EGD) and endoscopic ultrasound revealed a polypoid, heterogeneous, friable, solid 0.8-cm nodule arising from the second layer of the gastroesophageal junction. Similar nodules were also visualized in the gastric body. Biopsies of the gastric lesions demonstrated active chronic gastritis with expansion of the lamina propria by a chronic inflammatory infiltrate without granulomas. CMV inclusions were detected by both routine and immunohistochemistry (IHC) (Figure 1). A repeat EGD completed 7 weeks later for persistent symptoms demonstrated growth of the nodule to 1.3 cm with additional gastric nodules in the fundus measuring up to 1.5 cm and friable mucosa along the lesser curvature of the stomach (Figure 2). Repeat biopsies demonstrated ongoing severe active chronic gastritis with granulation tissue and purulent exudate consistent with ulceration and without granulomas. CMV was again demonstrated on IHC. On serology, CMV IgM was not reactive and serum CMV DNA was absent. Biopsies were negative for *H. pylori*, treponemal organisms, acid-fast bacilli, dysplasia, or malignancy.

**Figure 3.**
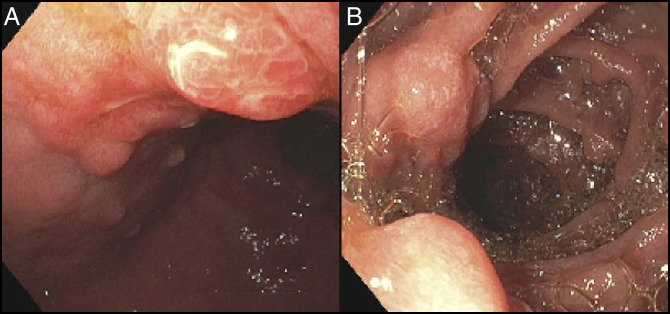
(A and B) Gastric polypoid lesion with the area of ulceration at the incisura and duodenal polypoid lesion with persistent inflammation.

**Figure 4.**
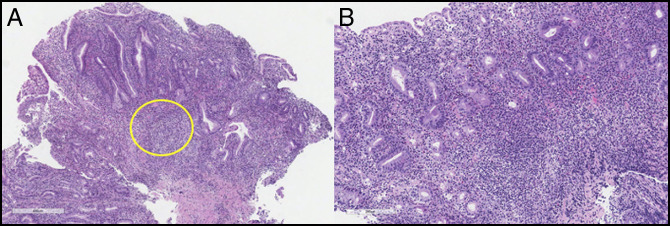
(A) Gastric mucosa (incisura) with well-formed granuloma (yellow circle) and extensive lymphoid and plasmacytic infiltrate within the lamina propria, along with reactive epithelial alterations. (B) Duodenal biopsy with duodenal mucosa with prominent lymphoplasmacytic infiltrate in the lamina propria, lymphocytes within the crypt epithelium, and reactive surface epithelial alterations.

The patient was treated with oral valganciclovir for 2 weeks after the first EGD. This was followed by intravenous ganciclovir for 6 weeks for unrelenting epigastric discomfort after the second EGD; subsequent EGDs for persistent pain demonstrated persistently inflamed polypoid lesions in the antrum and duodenum (Figure 3). Although repeat biopsies were negative for CMV by IHC, they demonstrated noncaseating granulomas, along with moderate-to-severe diffuse acute and chronic lymphoplasmacytic inflammation with lymphoid aggregates, cryptitis, and crypt abscesses (Figure 4). Investigations were negative for syphilis, fungal infections (such as coccidiosis and cryptococcus), and neoplasm.

**Figure 5.**
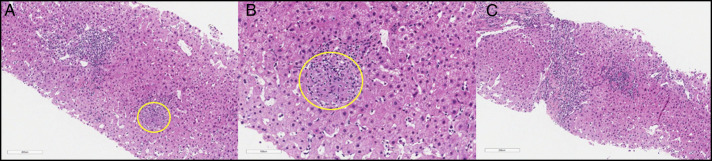
Liver biopsy showing (A) well-formed granulomata (yellow circle) with associated lymphocytes in the liver parenchyma, (B) the granuloma (higher magnification), and (C) portal-based lymphocytic inflammation in the remaining liver.

Ensuing investigations including thoracic and abdominal CT, CT enterography, and colonoscopy did not reveal any evidence of inflammatory bowel disease. There were multiple subcentimeter lymph nodes noted throughout the mediastinal, precordial, and mesenteric chains, as well as persistent splenomegaly of 21 cm, all of which were stable from previous imaging. A core needle para-aortic lymph node biopsy showed focal granulomas with epithelioid-type histiocytes. No clonal B-cell population was seen with gene rearrangement studies. Bone marrow biopsy was not pursued in consultation with hematology, and review of remote bone marrow biopsy performed 10 years ago was unremarkable. Given the history of cirrhosis, core needle biopsies of the liver were pursued. These demonstrated granulomatous hepatitis with bridging fibrosis (Figure 5). The patient was treated with a tapering course of prednisone beginning with 20 mg of prednisone daily for a presumed diagnosis of sarcoidosis, with resolution of her symptoms on steroids.

## DISCUSSION

Gastric sarcoidosis and intestinal sarcoidosis are rare conditions with 2.5% and 3.4% of systemic sarcoid patients respectively affected with a lack of clear pulmonary involvement being uncommon.^9,10^ Similarly, CMV reactivation in the intestinal tract is rare in immunocompetent individuals.^2^ Both CMV gastritis and gastric sarcoidosis present with nonspecific symptoms, such as abdominal pain, nausea, and vomiting. Clinical suspicion for CMV would be high in patients with immunosuppression (such as transplant patients or patients undergoing intense chemotherapy), whereas suspicion for sarcoidosis would be higher in patients with concurrent pulmonary symptoms and nongastrointestinal symptoms of sarcoidosis such as subcutaneous nodules.

The longstanding history of cirrhosis and lymphadenopathy suggests that this patient likely had pre-existing systemic sarcoidosis with initial subclinical pathology. With granulomatous changes seen on stomach biopsies, Crohn's disease, tuberculosis, autoimmune enteropathy or hepatopathy, sarcoidosis, syphilis, fungal infections such as coccidiosis and cryptococcus, neoplasm, and drug effect were all on the differential. It remains unclear if CMV gastritis preceded gastroduodoenal sarcoidosis or vice versa. Several possible explanations exist for this unique presentation. Immune dysregulation associated with systemic sarcoidosis or an unknown trigger could have led to CMV reactivation, followed by manifestation of sarcoidosis in the stomach and duodenum. In support of this hypothesis, sarcoidosis is believed to be associated with abnormal cell-mediated immunity involving CD4, CD8, and CD19 T-cells which may increase the risk of opportunistic infections.^11^ However, there has been only one reported case of CMV infection in sarcoidosis, where a female patient with pulmonary sarcoidosis was found to have fever, night sweats, left upper quadrant pain, and elevated liver enzymes with high CMV IgM titers and elevated CMV polymerase chain reaction viral load contributing to the diagnosis.^12^ Although pulmonary CMV disease was suspected in this case, viral inclusions on pathology was not confirmed.^12^ An alternative explanation may be that CMV infection may have exposed the presence of gastroduodenal sarcoidosis in our patient, with immunosuppression and inflammation induced by subclinical sarcoidosis allowing for CMV reactivation, possibly through mechanisms involving macrophage activation and tumor necrosis factor-α. In support of this hypothesis, tumor necrosis factor-α is involved in granuloma formation in sarcoidosis and is also an important cytokine for viral replication.^3,13,14^ Finally, it is possible, although less likely, that CMV infection unveiled a de novo presentation of sarcoidosis. Further studies are required to elucidate the association between these 2 diseases.

## DISCLOSURES

Author contributions: K. Leung and U. Khan wrote the manuscript. I. Teo provided the pathology images. P. James and J. McCurdy revised the manuscript. J. McCurdy is the article guarantor.

Financial disclosure: None to report.

Previous presentation: This case was presented at the Department of Medicine Research Day; June 15, 2017; Ottawa, ON, Canada.

Informed consent was obtained for this case report.
